# Exploiting nozzle geometry to predict resolution in extrusion-based bioprinting: mathematical modelling of a power-law fluid

**DOI:** 10.1098/rsos.250504

**Published:** 2025-11-12

**Authors:** Amy Victoria Tansell, Nasim Mahmoodi, Joseph Patrick Crolla, Rosemary Julia Dyson, Galane Jingxi Luo, Lauren Elizabeth Jane Thomas-Seale

**Affiliations:** ^1^ School of Mathematics, University of Birmingham, Birmingham, UK; ^2^ Department of Mechanical Engineering, School of Engineering, University of Birmingham, Birmingham, UK

**Keywords:** bioprinting, additive manufacturing, three-dimensional printing, empirical modelling, rheology

## Abstract

Extrusion-based additive manufacturing (AM) is a popular technique used in the fabrication of three-dimensional constructs. Owing to the nonlinear manner in which process parameters affect resolution and printability, the optimal combination remains platform and material specific. This study proposes a user-friendly, adaptable model to predict the diameter of a printed line of material through extrusion-based bioprinting. Exploiting the geometry of an arbitrary, axisymmetric nozzle and assuming a power-law fluid, the model generated determines a relationship between the printed filament diameter and the pressure drop, nozzle travel speed, nozzle geometry and material flow properties. Employing the model prior to printing enables engineers to restrict process parameter space and minimize the dependence on the current print-and-test methodology before an optimal combination of process parameters is determined. Two materials (a poly(vinyl alcohol)-based (PVA-based) hydrogel and Nivea Crème), two temperature conditions and three nozzle sizes were used for model validation, presenting good agreement with model predictions. When the shear-thinning property is included, the coefficient of determination, *R*^2^, is greater than 0.97. This model provides context and direction for future optimization-driven design research for this advancing manufacturing technology.

## 1. Introduction

Additive manufacturing (AM) is a rapidly advancing technology that presents far greater design freedom compared to traditional manufacturing methods, allowing optimization to drive design and generate more efficient parts [1,2]. Its technologies have been widely employed in tissue engineering and have adopted the name ‘bioprinting’ [3]. Bioprinting involves the assembly of three-dimensional, often patient-specific, tissue-like structures via accurate deposition of a bioink (a formulation compatible with cell culture, sometimes containing encapsulated cells, and suitable for biofabrication) in a layer-by-layer fashion. Bioprinting provides a promising trajectory for tissue replacement and may in time satisfy organ shortages, emphasizing the importance of research into the optimization of its design strategy [4].

**Figure 1.**
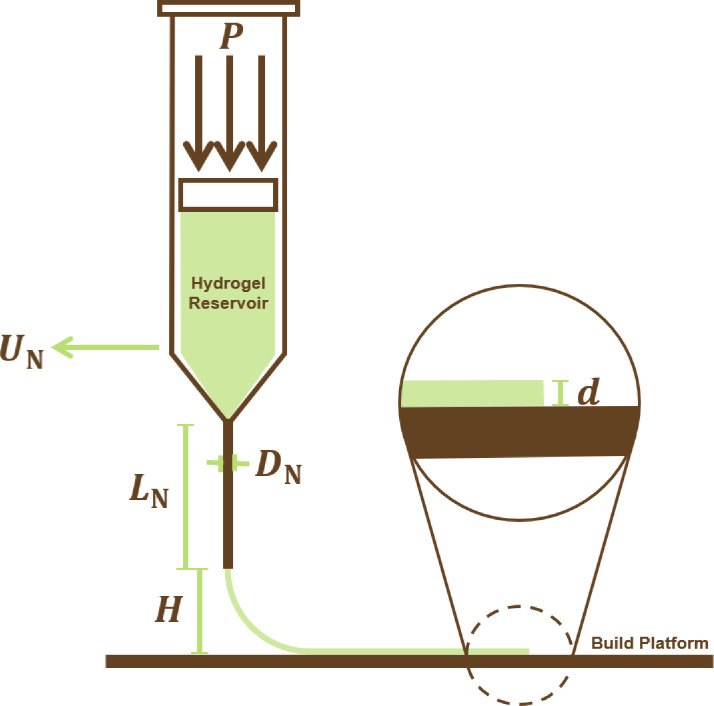
The extrusion-based bioprinting process, with P the pressure applied to the hydrogel, d the layer thickness and $D_{\text{N}}$, $L_{\text{N}}$, $U_{\text{N}}$ and H—the nozzle diameter, length, travel speed and offset, respectively.

Inspired by the established extrusion technique used in fused deposition modelling (a method of polymer AM), *extrusion-based bioprinting* involves continuous filaments of bioink that are pneumatically or mechanically extruded from a cartridge on to the build platform (as seen in Figure 1). Owing to its compatibility with a wide variety of viscosities and the high cell density that may be achieved within the three-dimensional structures produced, extrusion-based bioprinting is the most popular choice of bioprinting method described within the literature [5].

**Figure 2.**
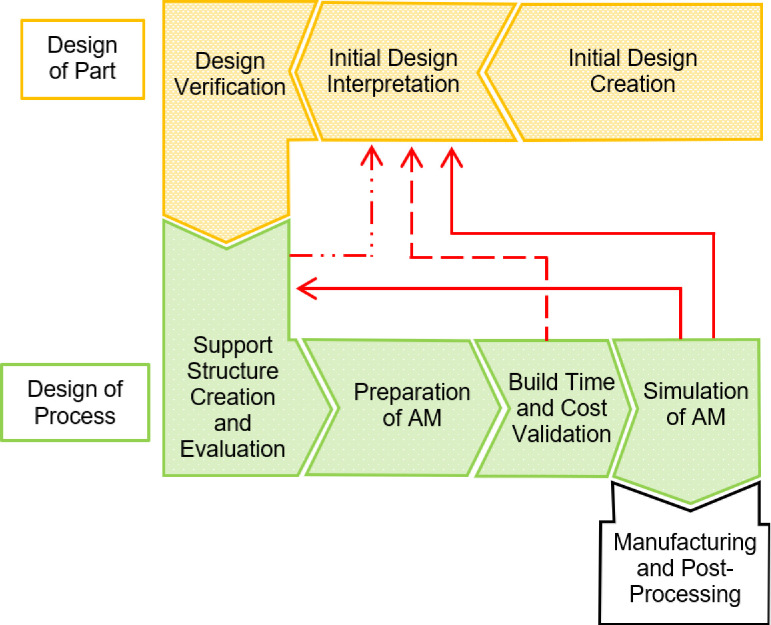
A flowchart presenting the current design for the AM workflow. Arrows (red) returning to earlier steps illustrate their current iterative nature.

Design for AM comprises the entire process chain for fabrication, from assessing the appropriate manufacturing method to preparing the geometric data and validating for a specific AM technology. To date, the design workflow remains fairly fragmented and time-consuming, presenting a sequence of manual steps repeated several times until the requirements for a particular component are met. Figure 2 outlines the part and process-specific design for AM workflow currently undertaken by design engineers (according to Wiberg *et al.* [6]), where the interdependent nature of part and process design is identified. A more comprehensive framework to guide fabrication can be found in Pradel *et al.* [7]. Automation of the workflow, with adaptable parametric computer-aided design models that synchronize with feedback loops, is necessary to enable designers to control the geometry and machine settings simultaneously and optimize the system as a whole.

Sustainable design is of utmost importance in the manufacturing industry. It is desirable to determine a methodology that generates minimal waste. Simulating the current iterative print-and-test methodology (seen in Figure 2) is a logical step towards achieving this aim. In this study, we focus on the initial preparation of the bioprinter, aiming to restrict process parameter space through prediction of the printed thread diameter and, thus, minimizing the dependence on the current print-and-test methodology to determine the optimal combination.

Although a consistent definition is yet to be established, we shall define the printability of a bioink to be the deviation of a printed construct from the initial three-dimensional model data for a particular application, characterized in terms of extrudability, shape fidelity and structural integrity [8]. Here, extrudability refers to the capability of a bioink to be continuously extruded through a nozzle in a controlled manner; shape fidelity to the difference in cross-sectional area of a filament from that set in the three-dimensional model data; and structural integrity to the capability of a construct to hold its intended structure post-print. Furthermore, when considering the viability of cells encapsulated in a bioink, the success of a construct is no longer just dependent on the printability of the material, but on the proportion of healthy cells remaining post-print [9]. In fact, owing to the complex rheological behaviour observed at the microscale, obtaining consistent results between applications has proven extremely challenging [10]. While the ultimate goal remains to optimize the printability of cell-laden materials, it is necessary to first understand fluids with similar rheological properties. As such, this paper primarily focuses on capturing fluid flow properties independent of cell concentration.

Future developments in the bioprinting field involve the generation of quantitative tools and methods to objectively analyse the performance of different bioinks during the extrusion-based bioprinting process. By simulating the process, any experimental analysis and between-laboratory biases can be greatly reduced, aiding the reproducibility of results across the literature, and increasing time and cost efficiency [11]. Approaches include mathematical models that use data from the rheological properties of a particular bioink as an input to predict the optimal printing environment for desired printability. These *in silico* models include power-law [12] (or Ostwald de Waele model [13]) and Herschel–Bulkley models [14]. The power-law model takes the form


(1.1) $$\tau =K{\dot{\gamma }}^{n},$$

where τ denotes shear stress, K denotes the consistency index (dependent on temperature, air pressure, shear rate and the flow behaviour index [15,16]), $\dot{\gamma }$ denotes shear rate and n denotes the flow behaviour index (with shear-thinning materials identified when 0<n<1 and Newtonian materials when n=1). We note that a Newtonian fluid presents a linear relationship between shear stress and shear rate, while fluids presenting a deviation from this linearity are termed non-Newtonian. An ideal bioink should exhibit a non-Newtonian behaviour: a shear-thinning property upon extrusion and rapid gelation upon deposition, to aid shape retention [17].

He *et al.* [3] identified the key process parameters necessary to gain precise control of the printability of a gelatin-alginate composite hydrogel via a series of experiments. They determined that the resolution of a printed line of hydrogel is affected by the *nozzle travel speed*, $U_{\text{N}}$, *nozzle offset*, H and *air pressure*, P. Schwab *et al.* [14] extended this shortlist by identifying a further dependence on the *geometry* of the nozzle. Their effect on the resolution of a print was examined theoretically (and validated experimentally using a Pluronic F-127 hydrogel) by Suntornnond *et al.* [18] via a mathematical model derived from the definition of dynamic viscosity, $\mu =\tau /\dot{\gamma }$. Although their work was based in a platform- and material-specific setting, the agreement between the theoretical and the experimental results emphasizes that mathematical modelling techniques pose a promising alternative to the existing methodology. The model presented in this study provides a generalization of that presented by Suntornnond *et al.* [18], where a straight nozzle is assumed.

Exploiting the geometry of an arbitrary, axisymmetric nozzle and assuming a power-law fluid, the aim of this study is to present a user-friendly, adaptable mathematical model to determine the printed filament diameter. In particular, a relationship between the printed filament diameter and the pressure drop, nozzle travel speed, nozzle geometry and material flow properties is determined. Examples of a conical and a straight nozzle are presented.

The model results are validated by using two distinctly different materials, a poly(vinyl alcohol)-based (PVA-based) hydrogel and the highly printable Nivea Crème, each using two different temperature conditions and three different conical nozzle sizes.

Employing the model prior to printing enables a ‘*window of printability*’ to be established, reducing the number of iterations of the current print-and-test methodology before an optimal combination of process parameters is obtained. The implications in the context of cell-laden materials are discussed.

## 2. Material and methods

### 2.1. Mathematical model

Conservation of mass is an important physical mechanism driving fluid flow; what leaves the nozzle must reach the build platform. As such, the foundation of the model is the concept of flux. We consider a viscous thread of material continuously extruded from a nozzle, with no-slip at the boundary between the fluid and the nozzle wall. We assume the thread maintains a circular cross section from the point of extrusion to the point it reaches the linearly moving build platform and assume the fluid flow to be steady and axisymmetric.

#### 2.1.1. Flux leaving the nozzle

Considering a small segment of an axisymmetric nozzle with an arbitrary cross-sectional area of height δz and radius r(z), the forces parallel to its centreline may be resolved, i.e.


(2.1) $$\begin{array}{lcr} & ({\text{F}}_{\text{p}}+{\text{F}}_{\text{ss}})\cdot n=0, & \end{array}$$

where ${\text{F}}_{\text{p}}$ is the pressure applied perpendicular to the cross section, ${\text{F}}_{\text{ss}}$ the frictional shear stresses along the nozzle wall and n the downward pointing unit vector. As such, we generate


(2.2) $$\begin{array}{lcr} & \delta P\pi r^{2}-2\pi r\;\delta z\;\sec\;\theta \;\tau \;\cos\;\theta =0, & \end{array}$$

with δP>0 the pressure drop (the difference between the pressure applied to the ink at the top and the pressure at the bottom of the segment), τ the shear stress (along the nozzle wall), θ the angle between the nozzle wall and the upward vertical and r the radius of the segment [19]. Here, the first term corresponds to the pressure drop across the cross-sectional area of the segment ($\pi r^{2}$), and the second, the component of shear stress parallel to the centreline (−τcos⁡θ) across its surface area (2πrδzsec⁡θ). Assuming a power-law fluid in a tube, where the shear rate is given by

(2.3) $$\dot{\gamma }=\frac{3n+1}{4n}\frac{4Q}{\pi r^{3}},$$

with Q the volumetric flow rate in the nozzle and n the flow behaviour index [20], equations (1.1) and (2.3) can be substituted into equation (2.2) for τ to obtain


(2.4) $$\begin{array}{lcr} & \delta P=\frac{2}{r}\;K{\left[\left(\frac{3n+1}{4n}\right)\frac{4Q}{\pi r^{3}}\right]}^{n}\delta z. & \end{array}$$

Integrating over the length of the nozzle, z, from 0 (at the top of the nozzle) to L (at the nozzle tip), generates


(2.5) $$\begin{array}{r} \Delta P=2K{\left(\frac{3n+1}{4n}\frac{4Q}{\pi }\right)}^{n}\int_{0}^{L}\frac{1}{r(z{)}^{3n+1}}\;dz, \end{array}$$

where $\Delta P=P_{\text{i}}-P_{\text{o}}>0$, with $P_{\text{i}}$ the pressure applied to the ink at the top of the nozzle and $P_{\text{o}}$ at the nozzle tip (assumed to be atmospheric pressure, with 1 atm = 101325 Pa). For a particular nozzle geometry, equation (2.5) can be employed to obtain the volumetric flow rate, Q.

**Figure 3.**
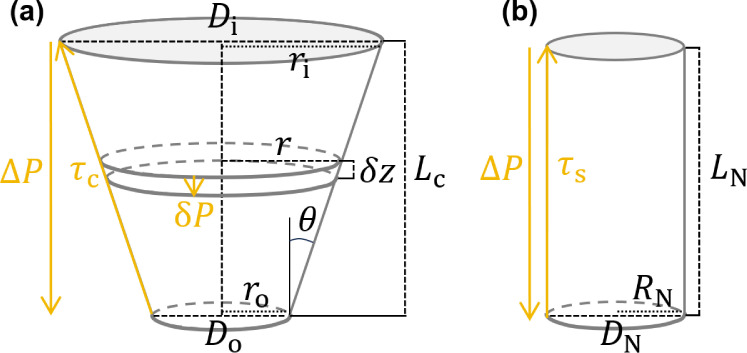
A schematic of (a) a conical nozzle with an inner diameter $D_{\text{i}}$ (inner radius $r_{\text{i}}$), outer diameter $D_{\text{o}}$ (outer radius $r_{\text{o}}$), length $L_{\text{c}}$ and an angle θ between the nozzle wall and the upward vertical, and (b) a straight, cylindrical nozzle with a diameter $D_{\text{N}}$ (radius $R_{\text{N}}$) and length $L_{\text{N}}$.r denotes the radius of an arbitrary small segment of the conical nozzle of height δz with a pressure drop δP; ΔP denotes the pressure drop between the pressure applied at the top and the pressure at the nozzle tip, and $\tau_{\text{c}}$ and $\tau_{\text{s}}$ the shear stress along the conical and straight nozzle walls, respectively.


Assuming a conical nozzle (seen in Figure 3a), equation (2.5) becomes

(2.6) $$\begin{array}{r} \Delta P=2K{\left(\frac{3n+1}{4n}\frac{4Q_{\text{c}}}{\pi }\right)}^{n}\int_{0}^{L_{\text{c}}}{\left(r_{\text{i}}+\left(\frac{r_{\text{o}}-r_{\text{i}}}{L_{\text{c}}}\right)z\right)}^{-(3n+1)}\;dz, \end{array}$$

where $r_{\text{i}}$ is the inner radius, $r_{\text{o}}$ the outer radius and $L=L_{\text{c}}$ the length of the conical nozzle, in the limits of integration. Solving and rearranging for $Q_{\text{c}}$, generates


(2.7) $$\begin{array}{r} Q_{\text{c}}=\frac{\pi }{4}\left(\frac{4n}{3n+1}\right)r_{\text{i}}^{3}r_{\text{o}}^{3}{\left[\frac{3n\tan\theta \Delta P}{2K\left(r_{\text{i}}^{3n}-r_{\text{o}}^{3n}\right)}\right]}^{\frac{1}{n}}, \end{array}$$

using $\tan\;\theta =(r_{\text{i}}-r_{\text{o}})/L_{\text{c}}$. Exploiting the definition of the volumetric flow rate, we obtain an average extrusion speed from the conical nozzle, ${\bar{u}}_{E_{c}}$, by dividing through by the cross-sectional area of the nozzle tip


(2.8) $$\begin{array}{rl} {\bar{u}}_{E_{c}}=\frac{r_{i}^{3}r_{o}}{4} & \left(\frac{4n}{3n+1}\right){\left[\frac{3n\tan\theta \Delta P}{2K\left(r_{i}^{3n}-r_{o}^{3n}\right)}\right]}^{\frac{1}{n}}. \end{array}$$

Following a similar methodology for a straight, cylindrical nozzle (seen in Figure 3b), the volumetric flow rate is given by


(2.9) $$\begin{array}{lcr} & Q_{\text{s}}=\frac{\pi R_{\text{N}}^{3}}{4}\left(\frac{4n}{3n+1}\right){\left(\frac{R_{\text{N}}\Delta P}{2KL_{\text{N}}}\right)}^{\frac{1}{n}}, & \end{array}$$

with $R_{\text{N}}$ and $L_{\text{N}}$ the radius and length of the straight nozzle, respectively, and an extrusion speed, ${\bar{u}}_{E_{s}}$, given by


(2.10) $${\bar{u}}_{E_{s}}=\frac{R_{N}}{4}\left(\frac{4n}{3n+1}\right){\left(\frac{R_{N}\Delta P}{2KL_{N}}\right)}^{\frac{1}{n}}.$$

#### 2.1.2. Flux on the build platform

The volumetric flow rate on the build platform takes the form


(2.11) $$\begin{array}{lcr} & Q_{\text{bp}}=\frac{\pi d^{2}\;U}{4}, & \end{array}$$

with d the printed filament diameter and U the build platform travel speed. Since flux at the nozzle tip and build platform must balance, equation (2.11) must equal equations (2.7) and (2.9). Equating equations (2.7) and (2.9) to equation (2.11) and rearranging for d, expressions for the printed filament diameters for conical and straight nozzles, $d_{\text{c}}$ and $d_{\text{s}}$, respectively, are generated:


(2.12a)dc={Di3Do38U(4n3n+1)[3nΔP4KLc(Di−DoDi3n−Do3n)]1n}12,(2.12b)ds=[DN38U(4n3n+1)(DNΔP4KLN)1n]12,

with $r_{\text{i}}=D_{\text{i}}/2$ and $r_{\text{o}}=D_{\text{o}}/2$, the inner diameter, $D_{\text{i}}$, and outer diameter, $D_{\text{o}}$, of the conical nozzle, respectively, and $R_{\text{N}}=D_{\text{N}}/2$ the diameter of the straight nozzle. Since an ideal bioink exhibits a shear-thinning property, we expect 0<n<1. In the Newtonian limit, where n=1 and K=μ, equation (2.12) reduces to


(2.13a)dc=[132μLcU(3Di3Do3Di2+DiDo+Do2)ΔP]12,(2.13b)ds=DN2(132μLNUΔP)12.

Note that equation (2.13a) becomes equation (2.13b) when $D_{\text{i}}=D_{\text{o}}=D_{\text{N}}$. In fact, equation (2.13b) is an expression exploiting Poiseuille’s law [21].

Assuming the nozzle geometry, build platform travel speed, U, and material properties, K and n, are known, equation (2.12) reduces to a proportionality, $d\propto (\Delta P{)}^{1/2n}$. With the syringe diameter being much greater than the nozzle tip diameter, we assume the pressure drop in the syringe to be negligible compared to that across the nozzle and, therefore, ΔP becomes, $\Delta P=P-P_{\text{o}}$, with P the air pressure applied to the hydrogel at the top of the syringe and $P_{\text{o}}$ at the nozzle tip. That is, $P_{\text{i}}$ can be assumed as P in the earlier definition obtained in equation (2.7). We may also replace U by the nozzle travel speed, $U_{\text{N}}$, without altering its meaning, enabling the use of the model across three-dimensional bioprinters. Whether the build platform travel speed guides the dragging of the filament along the build platform or the nozzle travel speed, the definition of the rate of fluid flow through an arbitrary cross section of the thread on the build platform remains the same.

### 2.2. Experimental methods

Two inks were used. The first, a PVA with molecular weight 146–186 kDa, hydrolysis of 99+%, and a 10% w/w concentration (Sigma Aldrich, St Louis, MO, USA) and the second, Nivea Crème (Beiersdorf Global AG, Germany), used as purchased. The PVA was dissolved in deionized water via mechanical stirring for 1 hour at $90^{\circ }$C. The hotplate was then removed and stirring continued for 1 hour, until the solution reached room temperature [22].

Nivea Crème is an example of a soft colloidal ink, maintaining a constant quality and composition [12]. Owing to its formula, it is temperature stable and able to withstand low and high temperatures without significant changes in its consistency [23]. As such, Nivea Crème is considered a highly printable material for use in bioprinting, presenting an ideal extrudability, shape fidelity and structural integrity. It has even been established as a demonstration ink by bioprinting pioneer REGENHU (Switzerland) [12]. On the other hand, the properties of PVA depend on its molecular weight and concentration as well as on temperature. PVA is a thermoresponsive material, behaving as a Newtonian fluid at room temperature; sub-zero temperatures initiate the non-Newtonian flow necessary for it to hold its shape. Choosing these two materials, a wide range of air pressures and two different build platform temperatures could be considered. This enabled the printing conditions required to achieve printability and emphasise the nonlinear manner to which process parameters affect it.

**Figure 4.**
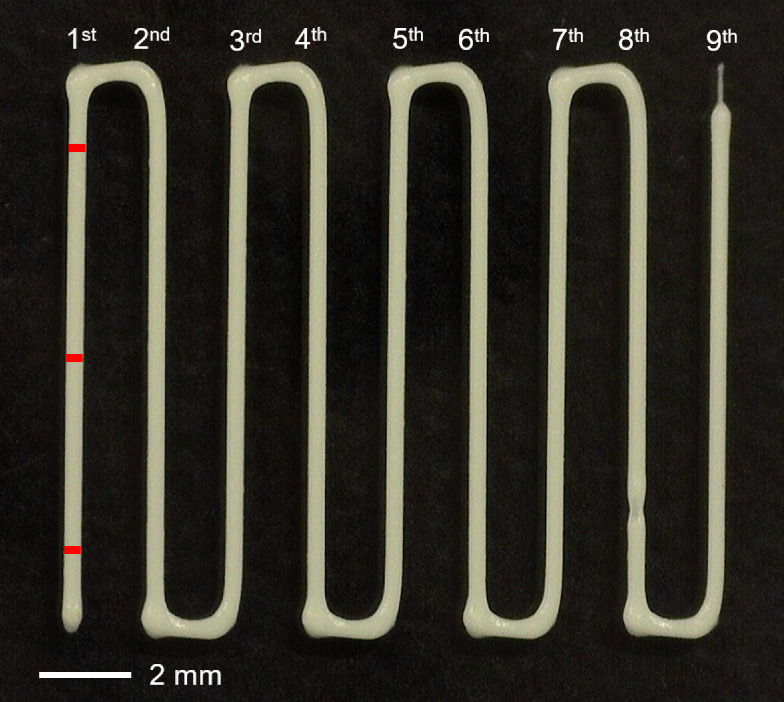
A print of the nine lines using Nivea Crème with a 22G conical nozzle, a 5 mm s^−1^ nozzle travel speed, 0.3 mm nozzle offset and an air pressure of $62\times 10^{3}$ Pa. The red lines marked on the first line indicate the three measurements taken before an average for the filament width was taken for each line.

Four experiments were undertaken using an INKREDIBLE+3D bioprinter (CellInk, Gothenburg, Sweden) with a 5 mm s^−1^ nozzle travel speed. As noted in §1, the experiments focused on process parameters, nozzle travel speed, nozzle offset, air pressure and nozzle geometry, with the aim of determining their effect on print resolution. The temperature of the build platform was also controlled in the PVA-based hydrogel case to enable filament shape retention (not being necessary for the Nivea Crème, the build platform varied with ambient temperature from approximately 21–28°C). For each set-up and material considered, nine lines of hydrogel were continuously printed on to the build platform, with each line measured at three points (start, middle, end) and an average taken, as seen in Figure 4. Note that with the accuracy displayed on the pressure gauge being to the nearest integer, close attention was paid to set it as close to the intended pressure as possible, that is, aiming for as close to $x\times 10^{3}$ Pa as opposed to $(x+1)\times 10^{3}$ Pa.

In experiment 1, the PVA-based hydrogel was printed using a frozen build platform ($-35\pm 1^{\circ }$C), a 25G conical nozzle (outer diameter: 0.25 mm) and a 0.3 mm nozzle offset, with nine different air pressures considered (10, 15, 20, 25, 30, 35, 40, 45, 50 × 10^3^ Pa). This sub-zero three-dimensional printing methodology is described further in Crolla *et al.* [22].

The next three experiments, 2(a), 2(b) and 2(c), were conducted with the Nivea Crème, exploiting its highly printable nature to determine the effect of nozzle geometry on shape fidelity, using a build platform at room temperature [12]. For 2(a), a 25G conical nozzle and a 0.1 mm nozzle offset were used, with 30 different air pressures considered ($67,68,69,...\;,96\times 10^{3}$ Pa); for 2(b), a 22G conical nozzle (outer diameter: 0.41 mm) and a 0.3 mm nozzle offset were used, with 18 different air pressures considered ($60,61,62,...\;,75,77,80\times 10^{3}$ Pa); and for 2(c), a 20G conical nozzle (outer diameter: 0.58 mm) and a 0.4 mm nozzle offset were used, with 17 different air pressures considered ($59,60,61,...\;,73,75,80\times 10^{3}$ Pa). For each air pressure, the print was repeated three times and an average line width was taken. With the nozzle travel speed fixed at 5 mm s^−1^, the nozzle offset was varied to enable adhesion at the range of nozzle tip diameters considered. Therefore, to limit the number of parameters changed across the Nivea Crème experiments, the ratio between nozzle tip radius and nozzle offset (the *aspect ratio*) was kept approximately the same throughout. Based on measurements taken by Gómez-Blanco *et al.* [24], the inner diameter remains the same across conical nozzle sizes and is taken to be 4.02 mm.

Each experiment was captured using a Dino-Lite Digital Microscope (AnMo Electronics Corporation, Taiwan) and analysed in ImageJ (NIH, MD, USA). All subsequent statistical analyses were performed in MATLAB${,}^{®}$ (MathWorks, MA, USA) v. 23.2.0.2380103 (R2023b). Regression analysis was conducted using the Optimization Toolbox^TM^, statistical hypothesis testing using Student’s *t*‐test in the Statistics and Machine Learning Toolbox^TM^, and a false discovery rate (FDR) correction made using the Bioinformatics Toolbox^TM^.

## 3. Results and discussion

**Figure 5.**
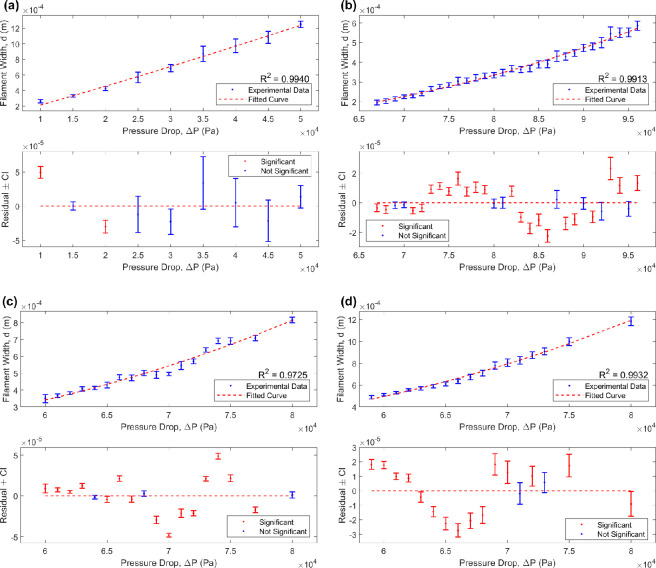
A regression analysis to determine the relationship between filament width (*y*-axis) and pressure drop (*x*-axis), with (a) a poly(vinyl alcohol)-based hydrogel and (b)–(d) Nivea Crème, with a 25G, 22G and 20G conical nozzle, respectively. In each case, the top graph exhibits a curve fitting of the form $d=ax^{1/2n}$, with a a constant coefficient and n the flow behaviour index, listed in Table 1. The residual plots (bottom) present the deviation between the data and fitted curve, with error bars representing 95% (uncorrected) confidence intervals and significance based on FDR-corrected *p*-values.

Fundamentally, the air pressure set on a bioprinter is the additional pressure necessary to overcome atmospheric pressure at the nozzle tip and initiate flow (relative to the viscosity of the medium). As such, air pressure and pressure drop are used interchangeably throughout this section. Figures 5a–d presents measurements for the average filament width, d, for a PVA-based hydrogel and Nivea Crème, respectively, using a variation of conical nozzle sizes and air pressures (also found in electronic supplementary material, Tables S1 and S2). Results from Student’s *t*‐test (with FDR correction) conducted on the residual between the experimental data and the model prediction are also presented (also found in electronic supplementary material, Tables S3–S6).

**Table 1.** Values of the constant coefficient, *a*, for the flow behaviour and consistency indices, *n* and *K* (Pa s^*n*^), respectively, corresponding to the optimal non-Newtonian curve fitting of the form *d = a*(Δ*P)*^1/2^*^n^*, for a poly(vinyl alcohol)-based hydrogel and Nivea Crème (using a variety of conical nozzles) obtained via regression analysis conducted in MATLAB**^®^** and captured in Figures 5a–d, with Δ*P* the pressure drop and *d* the printed filament width. The goodness of fit of each fitting is captured by the coefficient of determination, *R*^2^.

material and nozzle used	***a***	***n***	**K** (Pa s^*n*^)	***R* ^2^**
poly(vinyl alcohol)-based hydrogel, 25G	8.930 × 10^–9^	0.4570	2.9596 × 10^3^	0.9940
Nivea Crème, 25G	1.138 × 10^–18^	0.1694	11.09052	0.9913
Nivea Crème, 22G	1.220 × 10^–18^	0.1654	10.4465	0.9725
Nivea Crème, 20G	1.203 × 10^–18^	0.1635	9.2926	0.9932

### 3.1. Parameter fitting

An empirical model (equation (2.12a)) was derived, relating the printed filament diameter, d, to the pressure drop, ΔP, via a constant of proportionality, a, incorporating nozzle geometry (conical nozzle, $D_{\text{i}}$, $D_{\text{o}}$ and $L_{\text{c}}$, for experimental validation purposes), the hydrogel consistency index, K, the build platform travel speed, U (or nozzle travel speed, $U_{\text{N}}$) and the flow behaviour index, n. Figures 5a–d captures fittings to the experimental data for the PVA-based hydrogel and Nivea Crème, for the 25G, 22G and 20G conical nozzles, respectively, with functional form


(3.1) $$\begin{array}{lcr} & d=a(\Delta P{)}^{\frac{1}{2n}}, & \end{array}$$

where


(3.2) $$\begin{array}{lcr} & a={\left[\frac{D_{\text{i}}^{3}D_{\text{o}}^{3}}{8\;U_{\text{N}}}\left(\frac{4n}{3n+1}\right){\left(\frac{3n}{4KL_{\text{c}}}\frac{D_{\text{i}}-D_{\text{o}}}{D_{\text{i}}^{3n}-D_{\text{o}}^{3n}}\right)}^{\frac{1}{n}}\right]}^{\frac{1}{2}}, & \end{array}$$

as presented in equation (2.12a), with U replaced by $U_{\text{N}}$, since the INKREDIBLE+3D bioprinter relies on a moving nozzle. Table 1 lists the values of the constant coefficient, a, for the flow behaviour and consistency indices, n and K (Pa s${,}^{n}$), respectively, for each fitting.

#### 3.1.1. Exploiting the flow behaviour index

**Figure 6.**
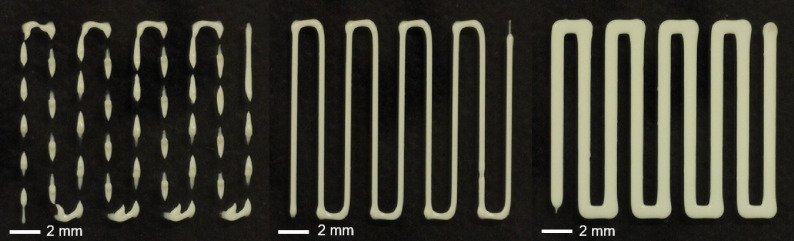
Three prints using Nivea Crème with a 22G conical nozzle, a 5 mm s^−1^ nozzle travel speed, and 0.3 mm nozzle offset, ranging from under- (left) to over-extrusion (right). An air pressure of $58\times 10^{3}$ Pa was used on the left, $62\times 10^{3}$ Pa in the middle and $80\times 10^{3}$ Pa on the right.

Across experiments, the data suggest that an increase in air pressure results in an increase in the average printed filament diameter, moving from an under-extruded state to an over-extruded state via the optimal air pressure, where the filament width, d, matches the nozzle tip diameter (as seen in Figure 6). In each case, the flow behaviour and consistency indices, n and K, respectively, were chosen to optimize the fitting to the data (the n and K corresponding to the minimum sum of squared residuals) and, from the residual plots, the data points are seen to lie randomly around the zero line (the fitted line), with an even distribution above and below, indicating that the model captures the data well (with goodness of fit, $R^{2}=0.9940$, 0.9913, 0.9725 and 0.9932, respectively, as seen in Figure 5). As seen in Table 1, the optimal n located in each case lies in the range (0,1), capturing the non-Newtonian, shear-thinning behaviour typical of materials used for extrusion-based bioprinting, with the Nivea Crème presenting a greater shear-thinning property compared to the PVA-based hydrogel, highlighting its highly printable nature [12]. Furthermore, n and K remain relatively similar (at an order of magnitude approx. $10^{-2}$) across the three Nivea Crème experiments, as expected, with differences potentially a result of experimental variation. In fact, since K=K(n), for the average $\bar{n}$ across these experiments, taken to be 0.1661, the value of K corresponding to the best fit in each case remains relatively similar (at an order of magnitude approx. $10^{-1}$), with K=10.5971,10.5473 and 9.6198 Pa s${,}^{n}$ for the 25G, 22G and 20G nozzles, respectively [16].

**Figure 7.**
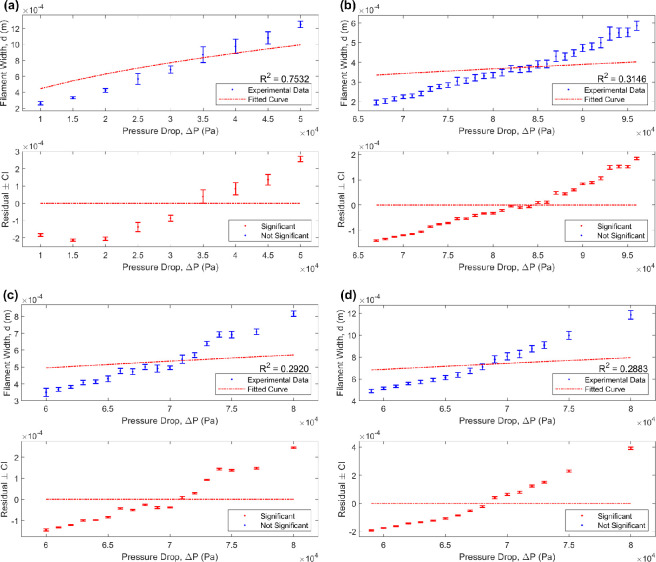
A regression analysis to determine the relationship between filament width (*y*-axis) and pressure drop (*x*-axis), with (a) a poly(vinyl alcohol)-based hydrogel and (b)-(d) Nivea Crème, with a 25G, 22G and 20G conical nozzle, respectively. In each case, the top graph exhibits a curve fitting of the form $d=ax^{1/2n}$, with n=1 and a a constant coefficient listed in Table 2. The residual plots (bottom) present the deviation between the data and the fitted curve, with error bars representing 95% (uncorrected) confidence intervals and significance based on FDR-corrected *p*-values.

Figures 7a–d presents the proportionality fitting for the Newtonian limit when n=1 (with optimal curve fittings listed in Table 2). Although it captures a similar trend to the data in the case of the PVA-based hydrogel ($R^{2}=0.7532$), it does not fit the shape closely, lying outside of the data towards the two end points (as seen in Figure 7a). In fact, across all cases, the residual plots reveal the model to match closely in the middle and then deviate from the data towards the two end points, presenting a statistically significant difference between the model prediction and experimental average across all points and indicating a poor fit overall. This reinforces the significance of n in the proportionality, capturing the non-Newtonian fluid flow behaviour revealed in the experimental data and, as such, obtaining a closer fit. This is reflected in Figure 5, where any statistically significant difference between the model and the experimental data is reduced as a result of implementing the non-Newtonian model.

**Table 2.** Values of the constant coefficient, *a*, for the consistency index, *K* (Pa s^*n*^), corresponding to the optimal Newtonian curve fitting of the form *d = a(*Δ*P)*^1/2^*^n^*, where *n* = 1, for a poly(vinyl alcohol)-based hydrogel and Nivea Crème (using a variety of conical nozzles) obtained via regression analysis conducted in MATLAB**^®^** and captured in Figures 5a–d, with Δ*P* the pressure drop and *d* the printed filament width. The goodness of fit of each fitting is captured by the coefficient of determination, *R*^2^.

material and nozzle used	***a***	**K** (Pa s^n^)	***R* ^2^**
poly(vinyl alcohol)-based hydrogel, 25G	4.448 × 10^–6^	2.2388 × 10^7^	0.7532
Nivea Crème, 25G	1.296 × 10^–6^	1.9014 × 10^6^	0.3146
Nivea Crème, 22G	2.020 × 10^–6^	1.0917 × 10^6^	0.2920
Nivea Crème, 20G	2.806 × 10^–6^	7.7960 × 10^5^	0.2883

In the PVA-based hydrogel and Nivea Crème experiments, a total of 27 and 81 filament width measurements were recorded per air pressure, respectively. With fewer measurements in the PVA-based hydrogel case, average values may be skewed by measurement error resulting in wider 95% confidence intervals compared to the Nivea Crème experiments, as seen in Figures 5a and 7a. As such, comparison of experimental averages with model prediction may be unreliable, with any difference lacking statistical significance. Therefore, repeating the PVA-based hydrogel experiments three times, as conducted with the Nivea Crème, may greatly improve its reliability and enable a greater statistical power.

#### 3.1.2. Practical use

**Figure 8.**
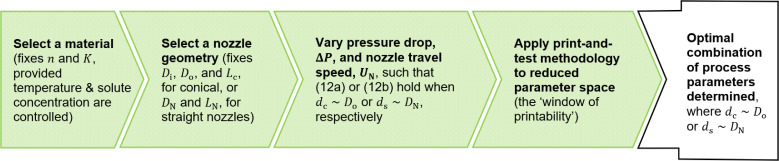
A flowchart presenting how the user may implement the model to establish a *window of printability* and reduce dependence on the current print-and-test methodology.

Presenting a good agreement with experiment, equations (2.12a) and (2.12b) can be implemented by the user to establish a *window of printability* and reduce the number of iterations of the current print-and-test methodology before an optimal combination of process parameters is obtained. Figure 8 presents a flowchart of how the user may apply the model in this way.

Since we require the printed diameter, d, to be as close to the nozzle tip diameter as possible, we may set $d_{\text{c}}∼D_{\text{o}}$ in equation (2.12a) and $d_{\text{s}}∼D_{\text{N}}$ in equation (2.12b), to some small tolerance. Then, fixing the material, n and K are specified (provided the temperature and solute concentration are controlled) while selection of a nozzle geometry provides $D_{\text{i}}$, $D_{\text{o}}$ and $L_{\text{c}}$, for conical, or $D_{\text{N}}$ and $L_{\text{N}}$, for straight nozzles. As such, equations (2.12a) and (2.12b) reduce to


(3.3) $$\begin{array}{lcr} & \frac{U}{\Delta P^{\frac{1}{n}}}∼A_{j}, & \end{array}$$

where j=c,s and


(3.4a)Ac=Di3Do8(4n3n+1)[3n4KLc(Di−DoDi3n−Do3n)]1n,(3.4b)As=DN8(4n3n+1)(DN4KLN)1n,

for conical and straight nozzles, respectively. Therefore, the pressure drop, ΔP, and build platform travel speed, U (or nozzle travel speed, $U_{\text{N}}$), may be varied such that equation (3.3) is satisfied to some small tolerance. In doing so, a *window of printability* is established, and the current print-and-test methodology is conducted on a reduced parameter space. By generalizing to an arbitrary, axisymmetric nozzle geometry, this methodology generalizes that implemented by Paxton *et al.* [12], where a straight nozzle is assumed, and enhances the applicability of the tool in the broader context of printing with cell-laden materials.

#### 3.1.3. Implications for cell-laden bioinks

At present, the principal objective for the model is to optimize printability, specifically, shape fidelity. However, as revealed in §1, in the case of cell-laden bioinks, the complexity of the optimization problem increases twofold. When cell-laden, a suitable trade-off between printability and cell viability has to be determined to achieve optimal mechanical and biological performance of the intended construct [25–28].

The *residence time* (how long a material particle is exposed to certain shear conditions [12]) and *shear forces* imposed on the cells need to be controlled. If they are too high, they can cause a loss in immediate and long-term cell viability [29], particularly for those cells travelling along the nozzle wall [12,30]. Temperature control can also be crucial to cell viability. Ouyang *et al.* [31] explored the biofabrication of embryonic stem cells into cell-laden constructs and discovered that control of the nozzle and chamber temperatures could lead to an increase in cell viability from 55.5 to 90%. In fact, the time period required for assembly and maturation of the cell-laden construct could even exceed the cell survival time [32]. On the other hand, there is evidence to suggest that the encapsulated cells can affect the rheological properties of the bioink and the chemical processes involved and, thus, correspond to a loss in printability, but the extent of these effects still remains elusive [8,14,33,34].

The model is agnostic about whether the material is cell-laden or not. Nevertheless, it provides the engineer with a shortlist of parameters to experiment with, from which they can choose the combination corresponding to the optimal trade-off between printability and cell viability. The model therefore reduces dependency on print-and-test methodologies. This reduction is particularly valuable when printing with cell-laden materials, owing to the costliness of cells [11]. Furthermore, by considering an arbitrary, axisymmetric nozzle, a nozzle geometry more desirable for printing with cell-laden materials may be specified.

#### 3.1.4. Exploring nozzle geometry

The importance of nozzle selection is emphasized throughout the literature [35]. Mohammed *et al.* [36] optimized the material and process parameters for a semisolid extrusion-based three-dimensional printing technique involved in the three-dimensional printing of drug products. They concluded that a straight nozzle requires a higher pressure with a greater chance of clogging, while conical nozzles require a much lower pressure, with chances of clogging greatly reduced, providing a much smoother, continuous flow from the nozzle tip. However, throughout the literature, a straight, cylindrical nozzle is typically assumed where empirical modelling techniques are employed [18,37–39]. For example, Suntornnond *et al.* [18] present a non-Newtonian extension of equation (2.13b), derived from the definition of dynamic viscosity, $\mu =\tau /\dot{\gamma }$ and assume a straight nozzle. They similarly find a direct proportionality between filament width and pressure drop but neglect the flow behaviour index, n, obtaining $d\propto (\Delta P{)}^{1/2}$. For the Pluronic F-127 hydrogel used, a good fit was obtained ($R^{2}=0.9962$) suggesting the material and its behaviour on the build platform could further affect the success of the fitting.

A straight nozzle was considered in the Nivea Crème experiments, but the minimum pressure necessary to extrude the material was extremely high, reducing the range of air pressures possible to explore on the bioprinter chosen. Furthermore, extrusion from the straight nozzle was relatively sporadic compared to the continuous flow achieved from the conical nozzles. As such, this nozzle geometry was excluded from the experiments.

To adjust to the experiments conducted, we specified a conical nozzle geometry in equation (2.12a), which presented a good agreement with the experiment, with $R^{2}>0.97$. But, ultimately, having considered an arbitrary, axisymmetric nozzle, a more general expression was established which can be adapted to any given axisymmetric nozzle geometry.

#### 3.1.5. Rheometric testing

Since the model is fitted to the data through the optimization of the flow behaviour index, n, and consistency index, K, it provides an efficient alternative to gain an understanding of the flow properties of the material. Typically, a rotational rheometer is used to measure the viscosity of a material as a function of shear rate, enabling the fitting of the power-law model to the linear region of the double logarithmic curve to determine n and K as follows:

(3.5) $$\ln\mu =\ln K+(n-1)\ln\dot{\gamma }.$$

Despite standard rheological tests being applied, the flow behaviour and consistency indices for Nivea Crème are seen to vary significantly across the literature (over an order of magnitude, as listed in Table 3), while for the PVA-based hydrogel used in this study, a value for these indices is unreported. These discrepancies suggest a high sensitivity to the rheometric testing conditions. The flow behaviour index determined from the curve fitting of equation (2.12) to experimental data sits within the range established in the literature. This suggests that the model could be capturing an acceptable estimation of the true flow behaviour (with additional information provided in the form of the shape fidelity expected). However, we emphasize that the experimental conditions and fitting methods employed will influence the data generated and, thus, affect the value of n and K obtained. Nevertheless, values determined through rheometric testing can be introduced into the optimization problem as the initial guess, reducing the number of iterations before a local minimum is located and increasing the chances of finding the values corresponding to the best fit. To validate these hypotheses, rheometric testing needs to be conducted in the future.

**Table 3.** Values of the flow behaviour and consistency indices, *n* and *K* (Pa s^*n*^), respectively, for poly(vinyl alcohol)-based hydrogels and Nivea Crème obtained via standard rheological tests.

study	materials	details	***n***	**K** (Pa s^*n*^)
Paxton *et al.* [12]	Nivea Crème	temperature: 24°C	0.552	26.1
Tu *et al.* [40]	Nivea Crème	temperature: unspecified	0.045	867
Jawad *et al.* [41]	PVA	5% w/w solution, with molecular weight, hydrolysis, and temperature unspecified	0.18	—
Lewandowska *et al.* [42]	PVA	10% w/w concentration, molecular weight 30–70 kDa, hydrolysis 87–90%, at a temperature of 30°C	0.98	0.037

#### 3.1.6. Model limitations and extensions

As a widely accepted model, the power-law model was used here, but it comes with its limitations. It is typically used to identify shear-thinning materials and, as such, is limited to a particular range of shear rates (generally 10^1^–10^4^ s${,}^{-1}$ [12]). Tu *et al.* [43] present a rheological characterization of Nivea Crème, highlighting the presence of a yield stress, $\tau_{0}$ (the minimum shear stress required to initiate flow). In fact, bioinks typically possess a yield stress, behaving elastically below and as a power-law fluid above this critical stress (a *viscoplastic* behaviour). With a focus on a power-law framework (when $\tau_{0}=0$), the model fails to capture this minimum shear stress, predicting flow even at very low shear stresses [44]. As such, for viscoplastic materials, the model may produce misleading results. While the power-law model may be sufficient for the prediction of suitable combinations of printing parameters (where only higher shear rates are relevant), for the purpose of optimizing shape fidelity, the Herschel–Bulkley model may be better suited [12]. To capture this yield stress behaviour, other models, such as the Herschel–Bulkley model, can be readily implemented into the proposed methodology. Georgiou [45] highlights that viscoplastic materials can slide even at shear rates below this critical rate. This suggests that the current no-slip condition between the nozzle wall and the fluid may need relaxing.

Owing to the nonlinear manner in which process parameters affect resolution, the parameters chosen in each case are unlikely to be the optimal combination. For example, on varying the air pressure, it is clear from the experimental results that there is an optimal choice (the point where the filament width, d, matches the nozzle tip diameter) of which above or below the material is over- or under-extruded, respectively (as seen in Figure 6), but, as the nozzle diameter increases, this optimal choice of air pressure is seen to decrease. Paxton *et al.* [12] suggest that the nozzle travel speed, $U_{\text{N}}$, should match the extrusion speed from the nozzle tip, but with its dependence on process parameters, such as air pressure, nozzle geometry, viscosity of the material (as captured theoretically in equations (2.8) and (2.10)) and temperature, the extrusion speed is rarely known in an experimental set-up. This emphasizes the necessity to return to the underlying physics of the system to derive a generalized theoretical mathematical model that enables a deeper understanding of how process parameters influence the complex rheological profile presented by a material [46].

Temperature control is crucial when bioprinting with thermoresponsive materials (such as the PVA-based hydrogel used in this study) since their sol–gel transition takes place at a certain temperature. Increased temperatures generally lead to a decrease in the viscosity of a bioink and, thus, correspond to a decrease in K and an increase in n; the fluid characterization approaches the Newtonian limit (n→1) [47]. A common methodology to capture this behaviour is via the expression of the indices as a function of temperature and is typically in the form of an exponential ratio of temperature (an Arrhenius-type relationship) [15,48]. Although this relationship is deemed suitable from a mathematical point of view, an inverse relationship of n with temperature, as presented by Abu-Jdayil *et al.* [49], may better reflect the Newtonian limit at high temperatures. Ultimately, however, the choice of function for n and K is heavily influenced by the material and set-up used [50]. Furthermore, the model neglects to capture any thread deformation at the nozzle tip, where the velocity profile adjusts from pipe flow in the nozzle to extensional free-surface flow (e.g. die swell). Although die swell is often observed when extruding with hydrogels, the problem remains complex and minimal research has been conducted to quantify this behaviour [51]. Recent advancements in fused deposition modelling may propose strategies for advancement in extrusion-based bioprinting in the future [52,53].

## 4. Conclusion

Although the optimal combination of process parameters still remains platform- and material-specific, quantitative tools are being developed to minimize any between-laboratory biases. A user-friendly, adaptable model has been derived to predict the resolution of a line of material via extrusion-based bioprinting with an arbitrary, axisymmetric nozzle. In particular, the relationship between the filament diameter and the pressure drop, nozzle travel speed, nozzle geometry and flow properties of the material is determined. A PVA-based hydrogel and Nivea Crème were extruded pneumatically using a variety of air pressures and conical nozzle sizes, and the printed filament diameter was measured. Model predictions presented a good agreement with the experimental data, particularly when the shear-thinning property was captured, with quantified errors arising owing to the assumptions made and process parameters considered. Exploitation of the model enables a *window of printability* to be established, reducing the current dependency on print-and-test methodologies before an optimal combination of process parameters is obtained. By devising mathematical models that exploit the non-Newtonian fluid flow driving the material through the system, we hypothesize that a deeper understanding will be achieved of how process parameters influence this complex rheological profile. The insight gained from undertaking this work has the potential to guide future research in the automation and optimization of design for extrusion-based AM.

## Data Availability

All data supporting the findings of this study are included within the article and any supplementary information [54]. Raw experimental data and MATLAB® code used for regression analysis: Dryad [55].

## Authors' Notes

The address of J P Crolla differs from that where the work was carried out, which is currently, High Temperature Research Centre, Ansty Park, Coventry CV7 9BF, UK.
